# Mortality salience effects and status consumption–A reasoned action approach

**DOI:** 10.1371/journal.pone.0350005

**Published:** 2026-05-28

**Authors:** Jolien Arendsen, Guido M. van Koningsbruggen, Marieke L. Fransen

**Affiliations:** 1 Department of Communication Science, Vrije Universiteit Amsterdam, Amsterdam, the Netherlands; 2 Behavioural Science Institute, Department of Communication Science, Radboud University, Nijmegen, the Netherlands; South Australian Health and Medical Research Institute Limited, AUSTRALIA

## Abstract

Prior research suggests that mortality salience (MS) can promote status consumption, yet it remains unclear how these effects are formed. To address this gap, we applied the Reasoned Action Approach, which provides a systematic framework to examine how MS may influence intentions to engage in status consumption. Study 1, a belief elicitation study, identified the set of salient beliefs related to status consumption. Study 2 revealed that MS influenced two out of 29 beliefs underlying status consumption identified in Study 1. Additionally, experiential attitude was a weaker predictor of the intention to engage in status consumption after MS. However, no effects of MS were observed on the intention to purchase status products or its determinants. Taken together, these findings suggest that, although MS may influence some underlying beliefs about status consumption, these changes are too weak to translate into meaningful differences in consumers’ behavioral intentions. By using the Reasoned Action Approach, the present research advances our understanding of how MS may or may not influence consumer intentions. It also suggests that MS’s influence on status consumption may be more limited than previously assumed.

## Introduction

Human beings possess a unique awareness of mortality, which can lead to existential anxiety. According to Terror Management Theory (TMT) [1], this anxiety can be managed by engaging in symbolic behaviors that affirm meaning, identity, and self-worth. One such coping mechanism is consumption—particularly status-driven consumption—which serves to reinforce cultural values and personal significance [2,3]. Indeed, reminders of death have been shown to increase the desire for conspicuous and luxurious products [4,5]. The heightened death awareness caused by the COVID-19 pandemic [6] offers a contemporary example of this phenomenon. During this period, luxury brands such as Prada and Louis Vuitton reported record-breaking sales [7]. Moreover, studies revealed a surge in impulsive purchasing behaviors, particularly of hedonic and luxury products [8] and found that pandemic-induced thoughts of mortality significantly increased indulgent consumption [9].

**Fig 1:**
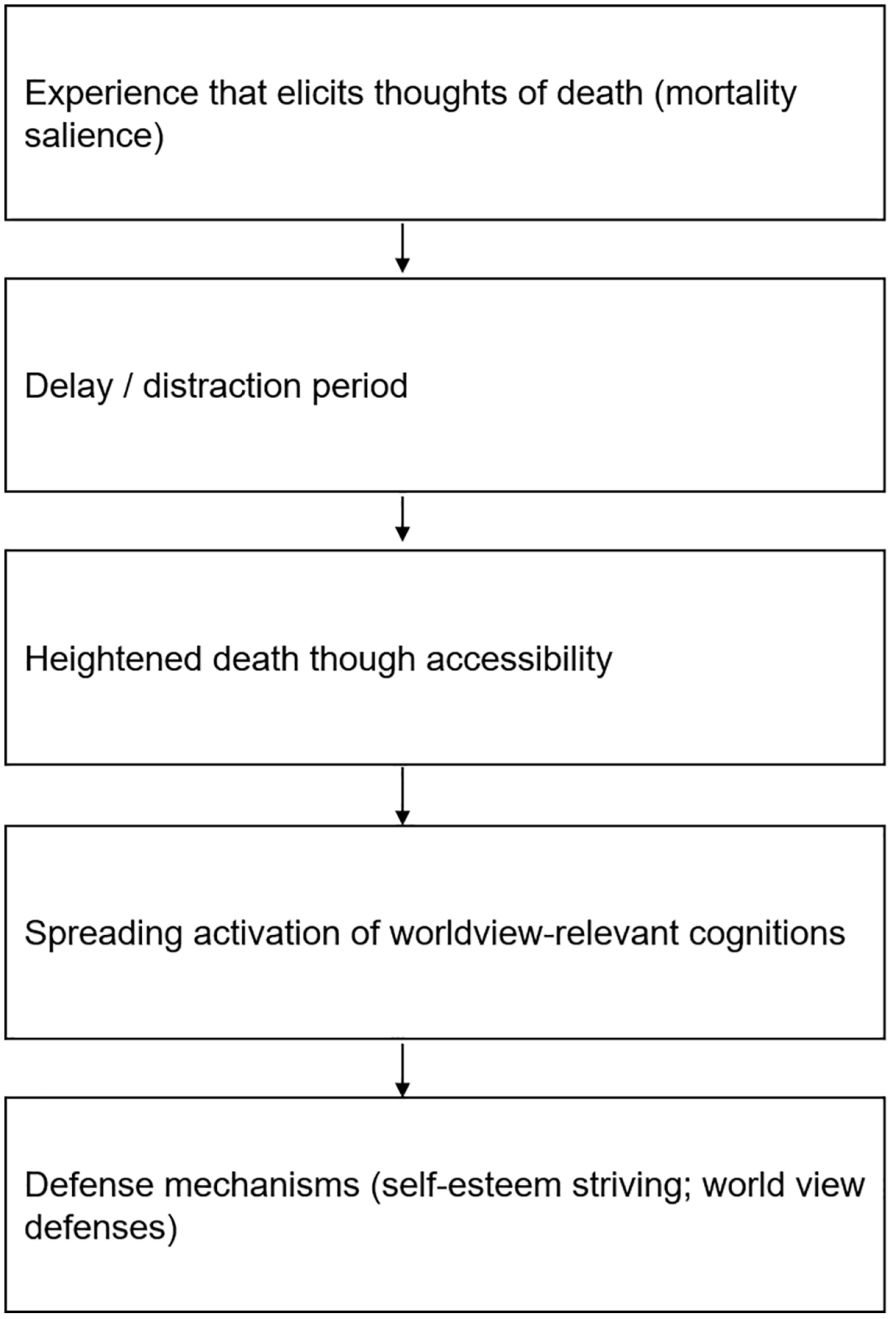
The process of terror management theory (adopted and simplified based on Kosloff et al., 2019).

This can be explained by the TMT-described mechanism through which humans cope with the fear of death, through a dual-component buffer consisting of cultural worldviews and self-esteem. Cultural worldviews provide a framework of shared beliefs about how the world operates and what gives life meaning, which provide a sense of order, purpose, and permanence. Within this framework, self-esteem functions as the subjective measure of how well an individual lives up to those cultural standards; when individuals meet these values, their resulting sense of personal worth acts as a shield against mortality-related anxiety. When faced with a reminder of death, and following a short delay or distraction, individuals experience heightened death-thought accessibility. This leads to a “spreading activation of worldview-relevant cognitions” [10], which is an activation of beliefs that are relevant to one’s worldview and self-esteem, which can subsequently trigger behaviors that bolster that worldview and self-esteem [11] (see Fig 1 for a conceptual overview). Consequently, behaviors that act as such a defense mechanism can be promoted by, so called, mortality salience (MS), which can be defined as “The awareness that life is finite and one’s death is inevitable” [12].

Various forms of consumer behavior can serve as a defense mechanism [12,13], specifically status consumption [2,14]. This stems from the assumption that money, products, and brands are important pillars of modern culture that contribute to people’s sense of self-worth [2]. According to TMT, MS will heighten the desire for money and products, especially those that convey status, as this makes people feel more valuable [4]. A recent meta-analysis [13] supports this notion, finding a small but positive and reliable effect of MS on consumer responses to status products (g = 0.28). However, the accompanying systematic literature review revealed mixed evidence and noted that these effects have not been systematically tested, and that the studies were often underpowered. Interestingly, many of the studies examined the direct effect of MS on status product consumption; implicitly assuming, but not empirically verifying, that intervening processes, such as cognitive and affective beliefs (e.g., beliefs related to worldviews and self-esteem), mediate this relationship. Furthermore, no studies to date have employed behavioral theories specifically designed to understand how factors like MS may affect status consumption. To address this gap, we use the Reasoned Action Approach (RAA) [15] to investigate whether and how MS influences the intention to purchase status products.

### The reasoned action approach and status consumption

The RAA is a well-developed theory based on decades of research. It is specifically designed to understand the factors that result in intention formation and subsequent behavior [16]. According to the RAA, the intention to perform a behavior in question is the single best predictor of behavior. Intention is shaped by three determinants: the attitude toward performing the behavior, the perceived norm, and perceived behavioral control. However, the importance and relevance of these determinants in predicting intention can vary across different target groups and behaviors. For example, status consumption may mainly be driven by perceived norm, while non-status consumption could be mainly driven by attitude. Each of the three determinants consist of two components. Attitude comprises an instrumental component (e.g., ‘Buying status products is good’) and an experiential component (e.g., ‘Buying status products is pleasant’). Perceived norm includes both an injunctive norm component (e.g., ‘People important to me think I should buy status products’) and a descriptive norm component (e.g., ‘People important to me buy status products’). Perceived behavioral control consists of an autonomy component (e.g., ‘It is up to me to buy status products’) and a capacity component (e.g., ‘I am able to buy status products’).

**Fig 2:**
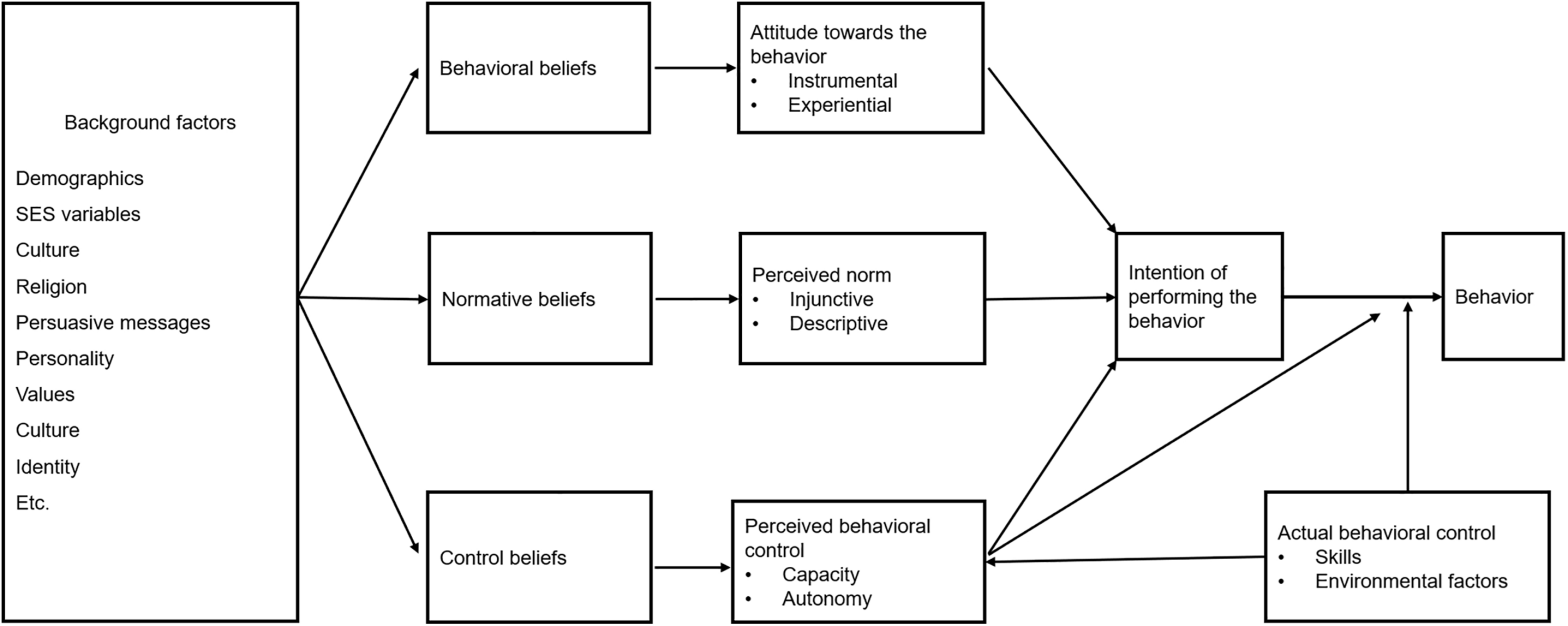
The reasoned action approach.

Attitude, perceived norm, and perceived behavioral control are general perceptions that people have about the behavior of, in this case, buying status products and are the result of specific beliefs about performing this behavior [15]. Attitude is shaped by behavioral beliefs, which reflect one’s thoughts and feelings about the likelihood and desirability of the consequences of buying status products. Perceived norm is shaped by normative beliefs, which reflect a person’s thoughts about what important others think and do regarding status consumption and the extent to which they want to conform to and be like those others. Perceived behavioral control is shaped by control beliefs, which reflect a person’s thoughts about facilitating or hindering factors for buying status products (see Fig 2 for an overview of the RAA). The three determinants—attitude, perceived norm, and perceived behavioral control—can be viewed as “cognitive summaries” of the complete set of specific behavioral, normative, and control beliefs [17]. According to the RAA, this means that intention and behavior are ultimately driven by specific beliefs that consumers have about the behavior [18].

**Fig 3:**
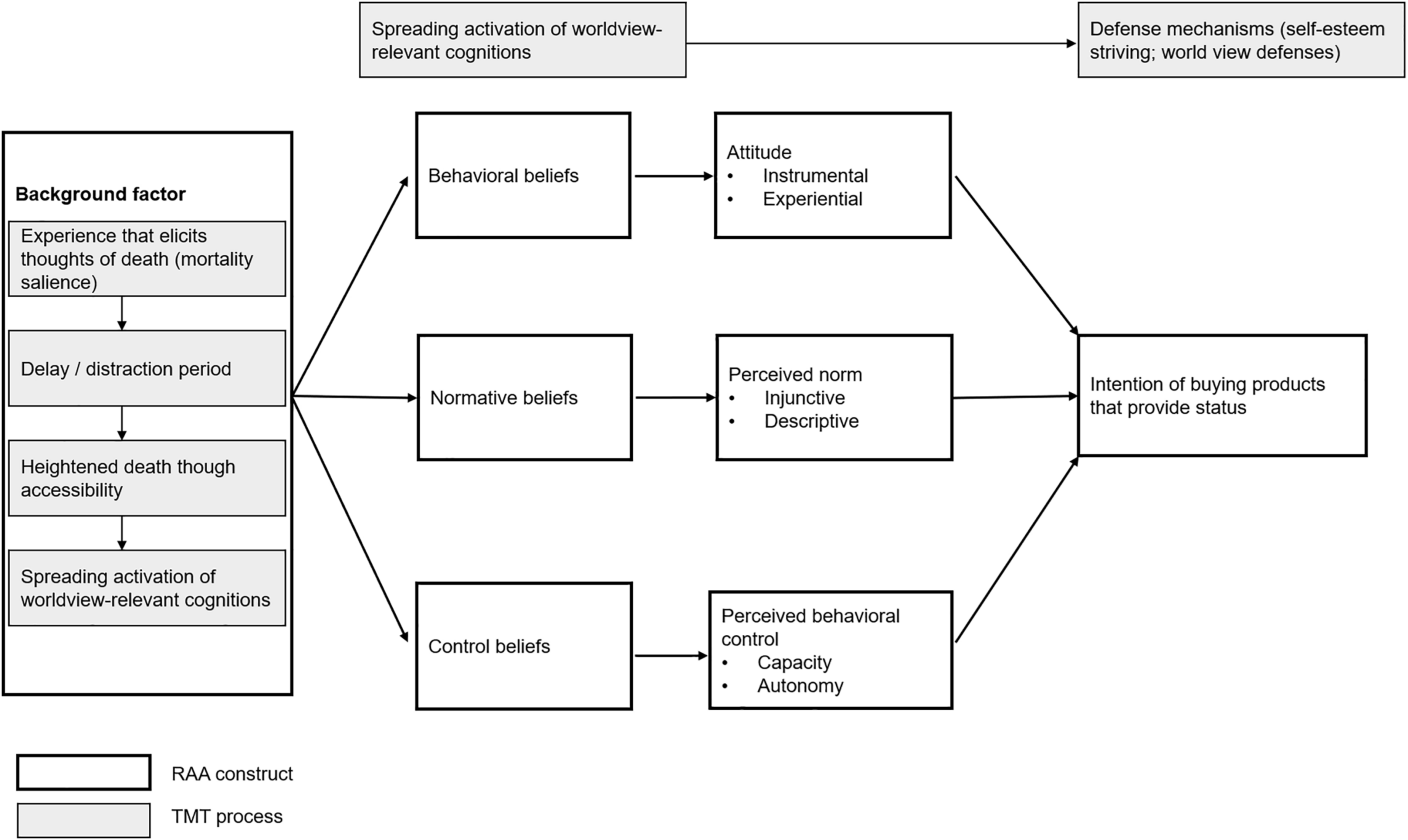
The reasoned action approach as applied to status consumption, including TMT processes.

Although people may have many different beliefs about a certain behavior, it is assumed that only the ‘salient beliefs’, which are the most highly accessible beliefs, at a given moment form the three determinants and subsequently influence intention and behavior. According to Fishbein and Ajzen [15], beliefs are formed through learning processes, such as direct observation (e.g., observing other consumers purchase status products), inference (e.g., concluding that obtaining status is possible by purchasing a certain product), and exposure to information (e.g., seeing advertisements for status products). Various background factors, such as experiences, information-exposure, or socioeconomic status (SES) variables, can influence belief formation and what beliefs are salient, thereby driving intention and behavior. Following the RAA logic, mortality salience could thus act as a background factor influencing consumers’ beliefs about buying status products and, consequently, their intention to purchase them. Thus, to increase our understanding of how MS influences the intention to buy status products, we should examine consumers’ beliefs about buying such products when mortality is (or is not) salient. Fig 3 provides an overview of the RAA model as applied to status consumption and TMT processes.

### How can mortality salience influence intentions of status consumption through RAA?

If MS increases consumers’ intentions to buy status products, as predicted by TMT and supported by a recent meta-analysis [13], the RAA framework may provide insight into how MS exactly influences this intention [15].

One possibility is that consumers with MS have different salient beliefs about buying status products than consumers without MS. For instance, people with MS may believe that consuming status products causes enjoyment, a belief that may not be salient among those who have not been exposed to MS. Another option is that MS and control consumers will have similar salient behavioral, normative, and control beliefs about buying status products, but that the strength of the relationship between those beliefs and the intention to buy status products may differ depending on MS status. That is, after MS, some beliefs related to buying status products could exert a stronger influence on the intention, that consequently could explain a difference in intention to buy status products between MS and control consumers. One final possibility is that experiencing MS changes the relative importance of the determinants that predict the intention to purchase status products. Under normal circumstances, the intention to buy status products is perhaps mainly driven by consumers’ attitudes toward buying status products. However, after MS, perceived norms may become more important in predicting the intention to buy status products. These differences could arise because, according to the RAA rationale, the relative importance of determinants predicting intention can vary between target groups (e.g., MS vs. control participants) [15].

In sum, using the RAA allows us to expand our understanding of how MS influences consumers’ intentions to purchase status products. By studying the beliefs underlying the purchase intention, we can determine exactly how MS influences consumers’ intentions and “where in the chain the influence breaks down” [15].

### The present research

As MS-induced status consumption is an often-pursued topic within TMT research and several studies suggest the existence of this effect (for an overview, see [13]), we aim to test the hypothesis that after MS (vs. control) participants will report a stronger intention to buy products that provide status to them (H1). Based on the assumption that MS functions as a background factor, and would expert it’s influence on intention indirectly (e.g., via beliefs) [15], we explore the potential influence of MS (vs. control) on participants’ attitudinal, normative, and control beliefs regarding buying status products (RQ1), and the relationships between the individual beliefs and the buying intention of the participant (RQ2). Furthermore, following Fishbein and Ajzen’s [15] reasoning that the importance of the determinants predicting the intention can vary across populations (MS vs control), we investigate whether the RAA determinants (instrumental/experiential attitude, injunctive/descriptive social norm, capacity/autonomy perceived behavioral control) influence the intention to buy status products differently in the MS and control condition (RQ3).

To test the hypothesis and answer the research questions, we will conduct two studies. Both studies will include a MS manipulation to examine the extent to which outcomes vary as a function of MS. Specifically, Study 1 uses a belief-elicitation procedure with open-ended questions to identify salient behavioral, normative, and control beliefs about buying status products. Study 2 uses a survey with closed-ended questions to determine which of these beliefs and RAA determinants guide participants’ intention to purchase status products. The results of Study 1 will inform the answer to RQ1, while the results of Study 2 will inform the answer to RQ1, RQ2, RQ3, and will be used to test H1.

Both studies were approved by the Research Ethics Review Committee of the Faculty of Social Sciences, Vrije Universiteit Amsterdam (Ref.No. 2023-11-7-542). Written informed consent was obtained digitally from each participant via a mandatory consent checkbox on the first page of the online questionnaire. Participation was anonymous, and no identifying information was collected. The second study was pre-registered (https://doi.org/10.17605/OSF.IO/PTXZK); full data and materials for both studies are available on OSF (https://osf.io/2m5yc/overview?view_only=1bf1ce1a24c64d36bbc0b731e716602e).

## Study 1

The aim of Study 1 was to identify behavioral, normative, and control beliefs about buying status products to answer RQ1 and for the development of measures for Study 2.

### Method

#### Participants and design.

The belief elicitation study employed a unifactorial design with two conditions (mortality salience vs. control). A total of fifty US adults (50% female) were recruited via Prolific and filled in the online survey. Ages ranged from 21 to 73 (*M* = 36.78, *SD* = 12.66) and educational levels varied between ‘high school diploma’ (12%) to ‘completed a PhD’ (4%), while the largest group of participants (42%) completed a bachelor’s degree. The data was collected on April 4^th^ 2024. Random assignment allocated 24 participants to the MS condition and 26 participants to the control condition (cf. recommendations of Fishbein & Ajzen [15] to include 15–20 participants for each subgroup). Chi-square and t-test analyses confirmed an even distribution of the measured demographic characteristics (gender, age, and education; *p* > .05 for all com*p*arisons), indicating that randomization was effective. Written informed consent was obtained from all participants, and they received £3.50 for their participation.

#### Procedure.

Standard belief elicitation procedures [15] were used and the survey consisted of three components. First, MS was manipulated by a writing task about their own death or a visit to the dentist, followed by a word puzzle, that functioned as a delay and distraction task to shift death-related thoughts from their conscious awareness to a state of high accessibility, a necessary condition for activating heightened death-thought accessibility [19]. Second, the topic of status consumption was introduced and described as follows: “We would like to know what you think and how you feel about the possibility of your buying products that provide status to you. When talking about these products that provide status, it can be about any kind of product (material possessions, food products, experiential products, etc.). What products give a sense of status can vary from person to person; for this study, it’s about products that provide you with status”. After this introduction ten open-ended questions were asked to elicit participants’ beliefs about status consumption. Third, participants were asked what kind of product(s) they had thought of when filling in the survey, followed by some demographic questions (gender, age, education level). Finally, participants were debriefed.

#### Manipulation and materials.

*Mortality salience* was evoked with the standard MS manipulation -by asking two open questions about participants own death: “Please briefly describe the emotions that the thought of your own death arouses in you” and “Jot down, as specifically as you can, what you think will happen to you as you physically die and once you are physically dead” [20] This manipulation has been proven effective in evoking distinct death responses. [21] In the control condition participants were exposed to a the standard control manipulation, by answering two questions about a dentistry visit: “Please briefly describe the emotions that the thought of your visiting the dentist arouses in you” and “Jot down, as specifically as you can, what you think will happen to you as you have a painful procedure done at the dentists office” [22]. Following previous research [23] we did not include a manipulation check, as this could in itself function as a mortality reminder.

*Behavioral beliefs* were asked through three open ended questions [15]: “What do you see as the advantages of your buying products that provide status to you?”, “What do you see as the disadvantages of your buying products that provide status to you?”, and “What else comes to mind when you think about buying products that provide status to you?”.

*Normative beliefs* were elicited with four open ended questions [15]: “Who (individuals or groups) do you think would approve or think you should buy products that provide status to you?”, “Who (individuals or groups) do you think would disapprove or think you should not buy products that provide status to you?”, “Sometimes, when we are not sure what to do, we look to see what others are doing. Who (individuals or groups) do you think are most likely to buy products that provide status to them?”, and “Who (individuals or groups) do you think are least likely to buy products that provide status to them?”.

*Control beliefs* were elicited with two open ended questions [15]: “What factors or circumstances would make it easy for you or enable you to buy products that provide status to you?” and “What factors or circumstances would make it difficult for you or prevent you from buying products that provide status to you?”.

#### Content analysis.

We followed the analysis procedures as described by Middelstadt [24]. First, two raters determined if the responses reflected behavioral, normative, or control, or no beliefs. Next, all responses within each of the three belief categories were categorized into a smaller number of distinct beliefs. This process employed an inductive and iterative approach, codes were developed during the review of participant responses. For example, in response to the behavioral belief question about the advantages of buying status products, a participant listed the following: “You may appear as you are wealthy and you have your life in place. People may treat you differently when you are out in public or in private. It could be a product that has quality and known to be great.”. These responses were initially coded as ‘perceived success’, ‘different treatment’, and ‘better quality’. After this initial coding, we (all three authors) discussed the semantic similarities between the codes. Through group consensus, we created clusters of related codes. For example, responses such as ‘higher quality’, ‘premium feel’, ‘name brand that fit better’, and ‘pieces that would last a life time’ were grouped under a code named ‘better quality’.

Next, we calculated belief frequencies for the whole sample as well as for each condition to rank-order the codes. We examined the belief frequencies and rank orders for each condition to determine if there were any differences (see the results below). Then, we identified the set of salient beliefs relevant to the entire sample to create the belief items for Study 2. When selecting these beliefs, we followed Fishbein and Ajzen’s [15] recommendations and chose as many frequently mentioned beliefs as possible, accounting for at least 75% of beliefs in each belief category. Following this rule ensured that all the most relevant beliefs were included without overlooking any.

### Results

The 50 participants listed a total of 781 responses. Of those, 91 responses were general statements that could not be included, such as “I don’t think about it” or “it’s silly”). This led to a total of 690 beliefs, that represented 18 different consequences (behavioral beliefs), 22 normative referents (normative beliefs), and 8 behavioral circumstances (control beliefs). All beliefs were rank-ordered by how many participants listed them, to come to the model set of salient beliefs we selected 12 consequences, 9 normative referents, and 5 control beliefs for further examination in Study 2. A total of 82% of the responses are included in these selected beliefs, thus meeting Fishbein and Ajzen’s [15] proposed rule of including at least 75% of responses. For a full overview of the responses, see Table 1.

**Table 1:** Frequency of listed beliefs for the full sample and per condition.

Behavioral beliefs	F total	F MS	F control	Normative beliefs	F total	F MS	F control	Control beliefs	F total	F MS	F control
**Positive or negative impression**	**49**	27	22	**Successful and rich people**	**48**	21	27	**Having plenty of money yourself**	**83**	42	41
**Not worth it**	**27**	14	13	**Family**	**30**	13	17	**Personal values**	**18**	11	7
**Expensive**	**25**	13	12	**Budget-minded or poor people**	**30**	14	16	**Having financial stability**	**15**	6	9
**Feel or think good about myself**	**17**	6	11	**People who care what others think**	**27**	14	13	**Judgement from others**	**13**	4	9
**Enjoyment**	**17**	9	8	**Status lovers**	**27**	21	6	**Availability and accessibility**	**10**	4	6
**Feel or think bad about myself**	**13**	8	5	**Friends**	**17**	10	7	Quality of the product	11	7	4
**Better quality**	**12**	8	4	**Insecure or lonely people**	**15**	8	7	Need for the product	7	3	4
**Attracts wrong people**	**11**	4	7	**Materialistic people**	**15**	9	6	Stability	7	4	3
**Connection**	**11**	7	4	**Partner**	**7**	3	4				
**Improve my life**	**10**	8	2	Old or young people	12	8	4				
**Wrong impression**	**8**	3	3	Peers	10	8	2				
**Better treatment**	**8**	4	4	Specific class	10	10	0				
Feel bad	7	2	5	Producers, marketeers, media	10	9	1				
Looking good	4	2	2	Unfriendly people	10	2	8				
Bad for the environment	4	2	2	Sustainable consumers	8	4	4				
Creates envy	2	2	0	Parents	5	1	4				
Limits experiences	1	1	0	People like me	4	2	2				
Vicious circle	1	0	1	Religious people	4	1	3				
				Strangers	4	2	2				
				Anyone	3	0	3				
				Trend setters	2	1	1				
				Friendly people	1	1	0				

Bold beliefs are selected as the modal set of beliefs and are included in study 2.

The most mentioned positive behavioral beliefs were about how status products buying results in others having a positive impression, that it results in enjoyment, and it gives a good feeling. On the negative side, behavioral beliefs revealed thoughts of expensive products, that might not be worth it, and getting a bad feeling about oneself. Families and friends were mentioned as the main groups of injunctive referents, while successful and rich people were mentioned as the main groups of descriptive referents. Beliefs about facilitating or hindering control factors were related to money, personal values, availability and judgement from others.

In the control condition participants listed 365 responses, while in the MS condition 325 responses were listed (respectively 80% and 83% of the responses were included in the selected beliefs). Between the conditions there were some differences in individual beliefs. Participants in the control condition more often thought that buying status products will improve their life, while participants in the MS condition more often believed it will result in them feeling good about themselves. Furthermore, in the control condition participants referred to people from a specific class as a referent group, while MS participants did not mention this group. Finally, the biggest difference can be found in the belief that ‘status lovers buy status products’, which was mentioned frequently in the control condition, and only a few times in the MS condition.

Lastly, we looked at the answers that participants gave to the question of what product(s) they thought of. There was a wide variety of answers and 6 participants (12%, all in the control condition) stated that they could think of no products that provide them with status. The most mentioned products were clothing, cars, housing, jewelry and electronics. There was no apparent difference between listed products in the two conditions.

Although there were small differences between conditions in how often specific beliefs were mentioned, when looking at all belief categories, they were similar across conditions, meaning that the same set of salient beliefs would be selected for both conditions. To summarize: in this study, it was found that MS and control participants hold similar behavioral, normative, and control beliefs about buying status products. This suggests that there is no clear influence of MS on the salient beliefs people have about buying status products. As described in the introduction, however, it could be that MS impacts the strength of specific beliefs or influences how these beliefs guide the intention to buy status products. This possibility will be investigated in Study 2.

## Study 2

The aim of Study 2 was to investigate how MS influence consumers’ beliefs, attitudes, perceived norms, perceived behavioral control, and intentions related to buying products that provide status. The beliefs selected from Study 1 were translated into survey questions, and all procedures were in accordance with RAA recommendations [15].

### Method

#### Participants and design.

The study employed a unifactorial design with two conditions (mortality salience vs. control). We conducted an a priori power analysis with G*Power to calculate the sample size needed to test our hypothesis (one-sided t-test; difference between two independent groups), with power = 0.95 and alpha = 0.05. We used an effect size of *g* = 0.28 (95%CI [0.03, 0.55]), based on the outcome of a previous meta-analysis [13]. With these parameters, G*Power suggested a total sample size of 554 participants. A total of 649 US participants were recruited via Prolific, between 12–17 June 2024, of which 590 completed the online survey. For all participants written informed consent was obtained and all participants received £3.50 for their participation. The responses of 27 participants were excluded, because they completed the experiment in less than five minutes (*n* = 8), failed the attention check (*n* = 15), or withdrew their permission to use their data (*n* = 4). The final sample consisted of 563 US adults (49.6% female, 47.8% male, 2.7% other) between the ages of 18 and 79 years old (*M* = 39.47, *SD* = 13.02). Educational levels varied between ‘no schooling completed’ (0.4%) to ‘completed a PhD’ (0.4%), while the largest group of participants (40.7%) completed a bachelor’s degree. Overall, the sample of Studies 1 and 2 are comparable. 284 participants were randomly assigned to the MS condition and 279 participants to the control condition. Chi-square and t-test analyses confirmed an even distribution of the included demographic variables (gender, age, and education; *p* > .05 for all comparisons), indicating that randomization was effective.

#### Procedure.

The procedure was similar to Study 1. Following informed consent participants were assigned to one of the two conditions and either answered two questions about death or about the dentist, followed by a word search puzzle as a distraction task. Then all RAA constructs were measured in the following order: intention, behavioral beliefs, attitude, normative beliefs, perceived norm, control beliefs, perceived behavioral control. Finally, participants were asked if anything else came to mind, and what kind of product(s) they had in mind when filling in the survey. Lastly, demographic questions (gender, age, education level) were asked, flowed by a debriefing.

#### Manipulation and materials.

*Mortality salience* was evoked exactly as in study 1, by asking two open questions about participants own death [20]. The two questions in the control condition were about a dentistry visit [21].

*Behavioral beliefs* were assessed, following Fishbein and Ajzen [15], by measuring outcome strength with twelve items on a seven-point scale (1 = very unlikely, 7 = very likely), items were formulated as “If I buy products that provide status to me, others will have a positive impression of me” or “My buying products that provide status to me, will give me pleasure”. Then, outcome evaluation was measured with twelve items concerning the same elements, such as “Others having a positive impression of me, is…” and “My having pleasure, is…” on a seven-point semantic differential (1 = bad, 7 = good). In line with RAA guidelines [15], for each belief, outcome strength and outcome evaluation items were multiplied and then a sum score for behavioral beliefs was calculated (*M* = 233.09, *SD* = 71.85).

*Injunctive normative beliefs* were assessed, following Fishbein and Ajzen [15], by measuring injunctive norm strength on a seven-point scale (1 = very unlikely, 7 = very likely) with three items formulated as “The following people are likely to think that I should buy products that provide status to me…” (e.g., partner; family; friends) and then motivation to comply with three items formulated as “When it comes to buying products that provide status to me, I want to do what my … think(s) I should do (Partner; Family; Friends). For each belief, injunctive norm beliefs and motivation to comply items were multiplied and then summed into a scale (*M* = 47.22, *SD* = 33.97).

*Descriptive normative beliefs* were assessed, following Fishbein and Ajzen [15], by measuring descriptive norm strength for nine referent groups (e.g., friends; materialistic people) formulated as “The following people are likely to buy products that provide status to them…” (1 = very unlikely, 7 = very likely), followed by the identification with the same referent groups, formulated as “When it comes to buying products that provide status to me, I want to be like…” (1 = strongly disagree, 7 = strongly agree). Each descriptive norm belief and identification with the referent group item were multiplied and then summed into a normative belief scale (*M* = 135.52, *SD* = 62.47).

*Control beliefs* were assessed, following Fishbein and Ajzen [15], by asking for the power of control factors, with five items on a seven-point scale (1 = strongly disagree, 7 = strongly agree), formulated as “Having … would enable me to buy products that provide status to me” (e.g., financial stability; plenty of money), followed by control strength of the same items, which was formulated as “When it comes to buying products that provide status to me, I will have…” (1 = strongly disagree, 7 = strongly agree). Each power of control factor and control strength item were multiplied and then summed into a control beliefs scale (*M* = 107.93, *SD* = 53.47).

*Instrumental and experiential attitudes* were measured with six seven-point semantic differential items, following Fishbein and Ajzen [15]; three for instrumental attitude (bad-good; unimportant-important; harmful-beneficial) and three for experiential attitude (unpleasant-pleasant; unsatisfying-satisfying; dull-exciting), see Table 2 for descriptives and reliability.

**Table 2:** Descriptive statistics for the whole sample.

*Correlations and descriptive statistics for the whole sample*										
	scale^a^	*M*	*SD*	1	2	3	4	5	6	7
1. Intention	.95	3.95	1.71	–						
2. Instrumental attitude	.90	4.17	1.59	.75**	–					
3. Experiential attitude	.93	4.76	1.66	.73**	.82**	–				
4. Injunctive norm	.80	3.65	1.60	.64**	.70**	.60**	–			
5. Descriptive norm	.68	3.75	1.58	.72**	.75**	.66**	.82**	–		
6. Autonomy -control	.75	5.94	1.19	.05	.01	.03	.05	.03	–	
7. Capacity -control	.75	4.58	1.68	.38**	.40**	.31**	.40**	.40**	.38**	–

^a^Scale reliability for intention and instrumental- and experiential attitude was calculated with a Chronbach’s alpha. The other measures each consisted of two items, so, following Eisinga et al. [25], reliability was calculated with a Spearman-Brown coefficient.**. Correlation is significant at the.01 level (2-tailed). All scales were measured with a 7-point scale.

*Injunctive and descriptive perceived norms* were assessed, following Fishbein and Ajzen [15], with four items on a seven-point scale (1 = strongly disagree, 7 = strongly agree), two for injunctive norms (“Most people whose opinions I value would approve of my buying products that provide status to me”, “Most people who are important to me think that I should buy products that provide status to me”) and two for descriptive norms (“Most people I respect and admire will buy products that provide status to them”, “Most people like me will buy products that provide status to them”, see Table 2 for descriptives and reliability.

*Perceived behavioral autonomy and capacity* were assessed, following Fishbein and Ajzen [15], with four items on a seven-point scale (1 = strongly disagree, 7 = strongly agree). Two items measured perceived autonomy (“It is mostly up to me if I buy products that provide status to me”, “I have complete control over my buying products that provide status to me” and two measured perceived capacity (“I am confident that I can buy products that provide status to me”, “It would be easy for me to buy products that provide status to me”, see Table 2 for descriptives and reliability

*Intention* was measured with three items on a seven-point scale (1 = strongly disagree, 7 = strongly agree) [15]: “It is likely that I will buy products that provide status to me”; “I expect that I will buy products that provide status to me”; “I want to buy products that provide status to me”, scores were averaged to form an intention scale, see Table 2 for descriptives and reliability.

### Results

#### Descriptive analysis.

Overall participants reported a moderate intention to buy status products (*M* = 3.95, *SD* = 1.71) and scores on the determinants were all medium to positive. As can be seen in Table 2, all determinants, apart from autonomy, show significant, moderate to strong, correlations with intention to buy status products.

Similar to study 1, we checked the product(s) people thought of while filling in the survey. Comparably, there was a wide variety of answers and some participants (8%) could not name any products that provide them with status (e.g., “I can’t think of anything I would get that could provide legitimate status to me.”). The most mentioned products were: cars, clothing, houses, jewelry, handbags. There were no apparent differences between the MS and control conditions in the types of products that were mentioned.

#### Registered analyses.

*Hypothesis 1.* To test whether participants after MS (vs. control) report a stronger intention to purchase products that provide status to them, we conducted an independent samples t-test with condition as the independent variable and intention to purchase status products as the dependent variable. The test showed no difference between the MS (*M* = 3.98, *SD* = 1.70) and control condition (*M* = 3.93, *SD* = 1.73), *t*(561) = −.35, *p* = .746, 95% CI[−.33, −.23], *d* = .03. Hypothesis 1 is therefore rejected.

*RQ1.* To answer what the influence of MS (vs. control) is on participants’ attitudinal, normative, and control beliefs regarding buying status products (RQ1), we ran independent samples t-tests with condition as IV and each belief (strength, evaluation, and product score) as DV. As can be seen in Table 3, for two of the 29 beliefs there was a small significant difference between the two conditions: in the MS condition participants had a stronger behavioral belief that they will “spend a lot of money” if they buy status products, and a stronger normative belief that “status lovers” buy more status products.

**Table 3:** T-test results: The effect of condition on behavioral, normative, and control beliefs.

Behavioral beliefs	*t*	*p*	*d*	MS condition (*n* = 284)		Control condition (*n* = 279)	
				*M*	*SD*	*M*	*SD*
Positive impression of me	1.17	.121	−0.10	26.46	11.11	27.53	10.50
Waste my money	.47	.321	−0.04	7.89	5.76	8.13	6.54
Spend a lot of money *	−1.84	.033	0.15	14.62	8.30	13.39	7.61
Feel good about myself	−.03	.490	0.00	25.98	11.80	25.957	12.00
Give me pleasure	−1.02	.153	0.08	27.12	11.89	26.10	11.75
Feel bad about myself	.31	.377	−0.03	8.06	7.11	8.26	7.93
Products of better quality	.12	.452	−0.01	27.86	10.84	27.97	10.99
Attract the wrong kind of people	−.35	.364	0.03	7.25	6.94	7.05	6.95
Networking and connection opportunities	−.88	.19	0.07	23.84	11.57	23.00	10.95
Improve my life	−.68	.25	0.06	23.52	11.46	22.89	10.89
Be perceived as richer than I am	.34	.366	−0.03	18.40	10.01	18.69	10.07
Be treated better by others	.62	.267	−0.05	22.80	11.37	23.38	10.94
**Injunctive normative beliefs**	***t***	***p***	***d***	**MS condition (*n* = 284)**		**Control condition (*n* = 279)**	
				***M***	***SD***	***M***	***SD***
Partner	−1.02	0.154	0.08	17.0	12.8	15.9	13.0
Family	−0.50	0.309	0.04	15.2	12.0	14.7	12.1
Friends	−0.76	0.225	0.06	16.2	12.1	15.4	11.6
**Descriptive normative beliefs**	***t***	***p***	***d***	**MS condition (*n* = 284)**		**Control condition (*n* = 279)**	
				***M***	***SD***	***M***	***SD***
Partner	−0.89	0.186	0.07	17.65	11.98	16.77	11.28
Family	−0.62	0.266	0.05	17.55	10.84	16.97	10.99
Friends	−0.76	0.223	0.06	19.50	10.53	18.82	10.61
Insecure people	0.23	0.411	−0.02	10.33	6.59	10.46	6.46
Materialistic people	−0.99	0.161	0.08	12.87	9.48	12.15	7.56
People who cannot afford it	0.00	0.500	0.00	8.98	7.48	8.98	7.47
People who care what others think	−0.92	0.179	0.08	13.66	9.16	12.99	8.18
Status lovers *	−1.97	0.025	0.16	15.70	11.70	13.88	10.23
Successful and rich people	−0.05	0.480	0.00	21.89	13.32	21.84	13.22
**Control beliefs**	***t***	***p***	***d***	**MS condition (*n* = 284)**		**Control condition (*n* = 279)**	
				***M***	***SD***	***M***	***SD***
Having financial stability	−1.02	0.154	0.08	24.68	13.00	23.55	13.35
Having plenty of money	−0.15	0.441	0.01	22.47	12.85	22.30	13.52
My personal values	−1.01	0.158	0.08	19.68	12.83	18.63	11.89
Opportunities (time and availability)	0.713	0.238	−0.06	21.69	12.33	22.44	12.40
Supportive social environment	−0.15	0.441	0.01	20.27	12.42	20.12	11.77

*. Effect is significant at the.05 level. All scales were measured on a 7-point scale.

*RQ2*. To determine the relationship between the individual beliefs and participants’ intention to purchase status products, we ran correlations between all beliefs and intention, for both conditions. As can be seen in Table 4, there was hardly any difference between the two conditions. The only noticeable difference was that in the MS condition the descriptive normative belief “insecure people buy status products” had a positive correlation with intention (*r* = .19), compared to a negative correlation in the control condition (*r* = −.14).

**Table 4:** Mean and standard deviation of behavioral, normative, and control beliefs and their correlations with intention, stratified per condition.

Behavioral belief	Full sample (*N* = 563)			MS condition (*n* = 284)			Control condition (*n* = 279)		
	*M*	*SD*	*r* with intention	*M*	*SD*	*r* with intention	*M*	*SD*	*r* with intention
Positive impression of me	26.99	10.81	.59**	26.46	11.11	.62**	27.53	10.50	.60**
Waste my money	8.01	6.15	.02	7.89	5.76	-.04	8.13	6.54	.04
Spend a lot of money	14.01	7.98	.26**	14.62	8.30	.26**	13.39	7.61	.23**
Feel good about myself	25.97	11.89	.72**	25.98	11.80	.73**	25.96	12.00	.75**
Give me pleasure	26.61	11.82	.71**	27.12	11.89	.72**	26.10	11.75	.75**
Feel bad about myself	8.16	7.52	-.08	8.06	7.11	-.03	8.26	7.93	.09
Products of better quality	27.91	10.90	.59**	27.86	10.84	.50**	27.97	10.99	.57**
Attract the wrong kind of people	7.15	6.94	.06	7.25	6.94	.039	7.05	6.95	.02
Networking and connection opportunities	23.42	11.26	.51**	23.84	11.57	.49**	23.00	10.95	.41**
Improve my life	23.21	11.17	.71**	23.52	11.46	.65**	22.89	10.89	.66**
Be perceived as richer than I am	18.55	10.03	.53**	18.40	10.01	.52**	18.69	10.07	.42**
Be treated better by others	23.09	11.15	.44**	22.80	11.37	.37**	23.38	10.94	.36**
**Injunctive normative belief**	***M***	***SD***	***r* with intention**	***M***	***SD***	***r* with intention**	***M***	***SD***	***r* with intention**
Partner	16.50	12.90	.56**	17.05	12.84	.57**	15.94	12.95	.56**
Family	14.91	12.01	.53**	15.16	11.96	.52**	14.66	12.08	.54**
Friends	15.82	11.86	.55**	16.19	12.13	.53**	15.43	11.59	.56**
**Descriptive normative belief**	***M***	***SD***	***r* with intention**	***M***	***SD***	***r* with intention**	***M***	***SD***	***r* with intention**
Partner	17.22	11.64	.51**	17.65	11.98	.52**	16.77	11.28	.49**
Family	17.26	10.91	.51**	17.55	10.84	.50**	16.97	10.99	.52**
Friends	19.17	10.56	.52**	19.50	10.53	.47**	18.82	10.61	.56**
Insecure people	10.40	6.52	.16**	10.33	6.59	.14*	10.46	6.46	.19**
Materialistic people	12.51	8.58	.35**	12.87	9.48	.33**	12.15	7.56	.37**
People who cannot afford it	8.98	7.47	.05	8.98	7.48	.03	8.98	7.47	.06
People who care what others think	13.33	8.69	.35**	13.66	9.16	.39**	12.99	8.18	.31**
Status lovers	14.80	11.02	.50**	15.70	11.70	.52**	13.88	10.23	.48**
Successful and rich people	21.86	13.26	.57**	21.89	13.32	.58**	21.84	13.22	.56**
**Control belief**	***M***	***SD***	***r* with intention**	***M***	***SD***	***r* with intention**	***M***	***SD***	***r* with intention**
Having financial stability	24.12	13.17	.51**	24.68	13.00	.57**	23.55	13.35	.46**
Having plenty of money	22.39	13.17	.43**	22.47	12.85	.43**	22.30	13.52	.43**
My personal values	19.16	12.37	.59**	19.68	12.83	.63**	18.63	11.89	.54**
Opportunities (time and availability)	22.06	12.36	.55**	21.69	12.33	.58**	22.44	12.40	.53**
Supportive social environment	20.20	12.09	.55**	20.27	12.42	.59**	20.12	11.77	.51**

*. Correlation is significant at the.05 level (2-tailed).**. Correlation is significant at the.01 level (2-tailed).

*RQ3.* To answer the question if the RAA determinants influence the intention to buy status products in the MS and control condition in a different way, we examined the regression weights in both conditions. See Table 5 for the results. In both conditions, the intention to buy status product was determined by the same set of RAA determinants, namely instrumental attitude, experiential attitude, and perceived descriptive norm. The injunctive norms and the capacity and autonomy PBC determinants did not significantly predict intention in both conditions. The relative importance of the determinants varied slightly across conditions. In the control condition, the experiential attitude was the most important predictor, while in the MS condition the instrumental attitude was most important. Perceived descriptive norm was the second most important predictor in both conditions. The experiential attitude coefficients were significantly different for the conditions: in the MS condition, experiential attitude became less important.

**Table 5:** Regression results: Intention regressed on attitudinal, normative and control variables stratified per condition.

	Full sample (*N* = 563)					MS condition (*n* = 284)					Control condition (*n* = 279)				
Explanatory variables	*M*	*SD*	β	*p*	VIF	*M*	*SD*	β	*p*	VIF	*M*	*SD*	β	*p*	VIF
Instrumental attitude	4.17	1.59	.23	<.001	4.28	4.21	1.64	.31	<.001	4.96	4.13	1.54	.15	.032	3.83
Experiential attitude*	4.76	1.66	.31	<.001	3.18	4.73	1.70	.22*	<.001	3.14	4.78	1.63	.41*	<.001	3.34
Injunctive norm	3.65	1.60	.03	.518	3.23	3.67	1.64	.10	.121	3.32	3.63	1.56	−.04	.557	3.21
Descriptive norm	3.75	1.58	.29	<.001	3.82	3.81	1.59	.26	<.001	3.90	3.68	1.57	.33	<.001	3.87
Perceived autonomy	5.94	1.19	−.00	.932	1.20	5.99	1.17	.04	.326	1.23	5.89	1.21	−.04	.360	1.18
Perceived capacity	4.58	1.68	.06	.044	1.49	4.68	1.66	.05	.268	1.58	4.48	1.69	.06	.162	1.43
*R*2			.65					.68					.64		

* Coefficients statistically significantly different between MS and control condition (determined via z-scores, which we obtained by dividing the difference between unstandardized coefficients by the root of the summed squares of their standard errors [26]). All scales were measured on a 7-point scale.

#### Additional analysis (not pre-registered).

Overall, the previous analyses revealed no significant differences in the effects of MS versus control on beliefs, nor in the relationship between beliefs and determinants of intention. To explore whether condition directly affects one of the six determinants of the intention to purchase status products, we ran ANOVAs with condition as the independent variable and the six determinants as the dependent variables. The results showed no significant differences for any of the variables (see Table 6).

**Table 6:** Anova results: Effect of condition on the six determinants.

			MS condition (*n* = 284)		Control condition (*n* = 279)	
Explanatory variables	*F*	*p*	*M*	*SD*	*M*	*SD*
Instrumental attitude	0.38	.539	4.21	1.64	4.13	1.54
Experiential attitude	0.10	.746	4.73	1.70	4.78	1.63
Injunctive norm	0.09	.767	3.67	1.64	3.63	1.56
Descriptive norm	0.91	.341	3.81	1.59	3.68	1.57
Autonomy -control	0.94	.333	5.99	1.17	5.89	1.21
Capacity -control	2.02	.155	4.68	1.66	4.48	1.69

All scales were measured on a 7-point scale.

## General discussion

The present research aimed to systematically investigate how MS influences status consumption by applying a theory developed for the prediction of behavior. Using the Reasoned Action Approach (RAA; [15]), we conducted two studies to determine whether MS affects people’s beliefs, attitudes, perceived norms and behavioral control regarding the purchase of status products.

Study 1 revealed participants beliefs about buying status products, highlighting thoughts about others’ impressions, key referent groups being friends and family, and control factors such as having enough money. There was no difference in the modal set of salient beliefs between the MS and control conditions. This suggests that, when faced with mortality salience, consumers have similar thoughts and feelings about buying status products as they do in situations where mortality is not salient.

Study 2 aimed to further investigate the beliefs and determinants of the intention to buy status products as a function of MS. The results firstly show no direct effect of MS on the intention to buy status products, thereby rejecting H1. To understand why MS did not have a direct influence on intention, it is necessary to analyze what happened at the level of beliefs. Therefore, we first looked how MS influenced the behavioral, normative, and control beliefs (RQ1) and found that there were limited differences: MS led to a stronger behavioral belief among participants that they will “spend a lot of money” if they buy status products, and a stronger normative belief that “status lovers” buy more status products. Subsequently, we looked at the relationships between the individual beliefs and intention (RQ2) and found that only the normative belief “insecure people buy status products” had a stronger correlation with intention in the MS condition. We further looked if the RAA determinants influenced the intention to buy status products differently in both conditions (RQ3). The findings show that after MS instrumental attitude becomes more important than experiential attitude, while in the control condition experiential attitude is more important. These findings show that there is a limited influence of MS on how the RAA determinants influence the intention to buy status products. Additionally, we explored whether MS had any direct influence on the six determinants, but no effects were observed. This means that, overall, there are no clear effects of MS on any of the RAA variables.

According to Fishbein and Ajzen [15], there can be several reasons why a background factor, such as MS, might not influence intention. One possibility is that MS and control participants hold comparable beliefs about buying status products. The results of Study 1 align with this possibility, as participants in the MS and control condition appeared to hold similar beliefs regarding buying status products. Another explanation is that even though MS and control consumers differ in a couple of beliefs, these differences may not be sufficient to result in different attitudes, perceived norms or perceived behavioral control, as these determinants are based on *sets* of salient beliefs. This possibility is a likely explanation for the pattern of results observed in Study 2, as only two of the 29 beliefs were impacted by MS, and neither correlated significantly with intention. Finally, another explanation put forward by Fishbein and Ajzen [15] is that even though the strength of instrumental and experiential attitudes in predicting the intention to buy status products varied between MS and control participants, this does not necessarily translate into a difference in intention if the behavior is also driven by other determinants. This could be the case here, as the intention of MS and control participants was also driven by descriptive norms.

### Theoretical implications for TMT

The results demonstrate an absence of a direct effect of MS on the intention to purchase status products, thereby rejecting Hypothesis 1. While this diverges from earlier empirical work [4,5] and a recent meta-analysis [13], the methodological rigor of this research directly addresses ongoing concerns regarding the robustness of MS effects. Recent reviews have noted that much of the TMT literature relies on underpowered, non-pre-registered studies with inconsistent dependent variables [13,27,28]. By utilizing a pre-registered design and a high-powered sample, we provide a rigorous empirical evaluation of TMT within the context of consumer behavior. The reporting of such null-results is critical for the integrity of the scientific record, as it guards against publication bias and the resulting artificial inflation of effect sizes in published literature [29].

Furthermore, our integration of the Reasoned Action Approach provides a granular explanation for this null-finding that previous studies lacked. Rather than simply failing to find an effect, our data suggest a specific theoretical boundary: MS may have insufficient influence on the behavioral, normative, or control beliefs that underpin status-driven consumption. This suggests that the psychological buffer provided by status products may be less universal than previously assumed. By documenting where effects do not occur, we can refine theoretical boundaries, ultimately fostering more reproducible and evidence-based science [30,31]. Consequently, the primary theoretical contribution of this work is the demonstration that future TMT research should move beyond simple direct-effect models and incorporate comprehensive theories of behavioral prediction, like the RAA, to gain a more nuanced account of how mortality salience functions within a consumer context.

### Limitations and recommendations

Although we took a systematic approach to studying the entire causal chain from beliefs to intentions, there could be other potential explanations for the lack of significant findings. One difference between our research and previous ones is that the latter often examined specific products, such as an apartment [32] or a watch [4], or luxury brands, such as a Mercedes car [33] or Louis Vuitton accessories [34], while we examined status consumption at the phenomenon level. It is possible that for specific products, specific beliefs become salient that might impact (determinants of) intention. However, products that were mentioned by our participants seem similar to products used in previous studies and included houses, cars, and high-end fashion. Our results show that, while there was a great variety of products people associated with status, there seems some consensus of those types of products. In this context, it is interesting that previous studies found MS effects on one specific product because the cultural worldview of each individual can differ, making it is implausible that everyone sees a car as a status symbol [35]. This is precisely why we chose to study status consumption at the phenomenon level: to ensure that the status products considered are relevant to each person’s own worldview. Nonetheless, future research could incorporate TMT and RAA with a focus on specific products.

When asked about descriptive normative beliefs, that is, the people or groups that buy status products, participants mentioned lonely, insecure, materialistic, and inauthentic people. It is likely that these groups are associated with buying status products for a reason and could be more susceptible to the effects of MS on status consumption [36]. However, in this study, we did not measure personality traits. Therefore, it remains possible that our sample, on average, scores low on traits typically associated with status consumption. However, because the focus of our research was to uncover how mortality salience works by using the RAA in combination with TMT, we have not measured personality traits nor analyzed potentially moderating influences of demographic characteristics. Randomization checks revealed no differences between conditions regarding age, gender, or education level, making it unlikely that these factors explain the current results. Nevertheless, for future research, it could be interesting to further study the role of demographic factors such as age [34,37] or cultural background (for example, focusing on the difference between “indulgent and restraint cultures” [38]), as well as personality measures, such as materialism [39,40] and self-esteem [41,42]. Given that a wide variety of moderating factors can influence mortality salience effects (see [13,43]) this remains a relevant direction for future research when investigating MS in the context of consumer decision-making.

The present study used the RAA to focus specifically on status consumption. However, the RAA may also help determine whether and how MS affects other types of consumer behavior. For instance, it could be used to examine prosocial responses, such as donation behavior [44,45], or in-group/out-group responses, such as the preference for domestic over foreign products [46,47]. To systematically test MS effects further, future studies should combine TMT with theories of behavioral prediction, while focusing on different consumer behaviors.

## Conclusion

Overall, this research does not support the idea that MS leads to increased status consumption. The results further cast doubt on the robustness of this proposed effect [13,27]. Even if mortality salience affects some consumers’ beliefs regarding status consumption, the current results suggest that these effects are too weak to cause meaningful differences in consumers’ intentions. To systematically investigate the effects of MS on consumer behavior, we propose that TMT research should benefit from theories of behavioral prediction. This approach could lead to a better understanding of both effects and null findings.
